# Age-Related Frailty: A Clinical Model for Geroscience?

**DOI:** 10.1007/s12603-020-1491-4

**Published:** 2020-10-13

**Authors:** Catherine Takeda, D. Angioni, E. Setphan, T. Macaron, P. De Souto Barreto, S. Sourdet, F. Sierra, B. Vellas

**Affiliations:** 1 Gérontopôle, Department of Geriatrics, CHU Toulouse, Cité de la Santé, Place Lange, 31059, Toulouse cedex 9, France; 2 Inserm UMR, 1027, Toulouse, France; 3 University of Toulouse III, Toulouse, France; 4 Saint George's Hospital, Beyrouth, Lebanon

**Keywords:** Frailty causes, aging, age-related frailty, frailty related to diseases, geroscience

## Abstract

In their everyday practice, geriatricians are confronted with the fact that older age and multimorbidity are associated to frailty. Indeed, if we take the example of a very old person with no diseases that progressively becomes frail with no other explanation, there is a natural temptation to link frailty to aging. On the other hand, when an old person with a medical history of diabetes, arthritis and congestive heart failure becomes frail there appears an obvious relationship between frailty and comorbidity. The unsolved question is: Considering that frailty is multifactorial and in the majority of cases comorbidity and aging are acting synergistically, can we disentangle the main contributor to the origin of frailty: disease or aging? We believe that it is important to be able to differentiate age-related frailty from frailty related to comorbidity. In fact, with the emergence of geroscience, the physiopathology, diagnosis, prognosis and treatment will probably have to be different in the future.

## Introduction

Over the past decades, frailty has commonly been accepted by the scientific and clinical communities (1, 2). Frailty is a clinical condition characterized by an excessive vulnerability of the individual to endogenous and exogenous stressors (3). In 2001, Fried et al. (4) established the Frailty phenotype as a clinical syndrome based on the presence of three out of five criteria: unintentional weight loss (4.5 kg in the past year), self-reported exhaustion, weakness (grip strength), slow walking speed and low physical activity. Rockwood and Mitnitski introduced a Frailty Index, which is based on an accumulation of age-related deficits. In their model, frailty is a continuous score summing signs, symptoms, disabilities and diseases (5) (6). The frailty process appears to be a transitional state in the dynamic progression from robustness to disability (7). Many factors leading to frailty have been identified: these include sociodemographic factors (e.g. older age, ethnical background, lower socioeconomic status), physical factors (e.g. obesity, ADL), psychological factors (depressive symptoms) (1), acute events or chronic disease (9).

In everyday clinical practice, we see older people becoming frail without any convincing clinical explanation, especially in the oldest-old. Frailty appears to be related to advancing age, what we can call “age-related frailty”. Despite many studies on frailty and its outcomes (10, 11, 12, 13, 14, 15, 16, 17, 18, 19), there remains considerable uncertainty as to the cause(s) of frailty (2, 20). Because of new insights provided by the emerging field of geroscience, it appears increasingly pertinent to differentiate age-related frailty from frailty related to comorbidity, since the physiopathology, diagnosis, prognosis and treatment will probably be different. In 2003, Fried et al. suggested the terms “primary” and “secondary” frailty to describe respectively frailty in the absence and presence of comorbidities (21). Fedarko hypothesized that the biology of frailty differed from normal age-related changes and other age-related diseases (22), underlying the importance of distinguishing different clinical Models. Further investigation is needed to validate the origins of frailty from a biological point of view. Geroscience, a new interdisciplinary field that aims to understand the relationship between the biology of aging and the biology of age-related diseases (23), thus has a critical role to play in identifying biomarkers of frailty (24) in order to define the origins of frailty.

The objective of this viewpoint is to underline the importance of distinguishing frailty related to age (25) from frailty related to comorbidity (26).

## Age-related frailty

Among a European population of persons aged over 65 years, 18% were reported to be frail (95% CI 15% to 21%) (27). The prevalence of frailty increases with age (4), and this is commonly believed to be related to biological age rather than chronological age (28)(29)(30)(31). Hoogendijk proposed frailty as an extreme consequence of the normal aging process (32) according to several longitudinal studies (33, 34).

In 2011, Fedarko defines aging as the decline and deterioration of functional properties at the cellular, tissue and organ level (22). Frailty manifests itself as age-related, biological vulnerability to stressors and decreased physiological reserves, resulting in a limited capacity to maintain homeostasis (35, 36). According to Fulop et al., frailty is a nonspecific state of vulnerability, which reflects multisystem physiological changes that do not always lead to disease, so some very elderly become frail without a specific life-threatening illness (37). The cellular responses of apoptosis, senescence, repair and the systemic response of immune activation/inflammation contribute to aging. In addition, aging reflects how damage propagates through a complex network of interconnected elements (38). These responses, together with changes in homeostatic mechanisms, are likely candidates for pathways that may contribute to the failure seen in frailty (22).

What is still unclear is whether this “age-related frailty” is just the consequence of the aging process in the oldest-old, or rather the consequence of several infra-clinic diseases (e.g. pre-diabetes, elevated blood pressure, limited arthrosis) that progressively lead to frailty. Of course, age-related frailty could be the result of the interactions between these pre-clinical states and the aging process. The main question that remains to be solved is whether it is possible to isolate age alone from the other possible causes of frailty, and thus to propose a model of age-related frailty that could contribute to further discoveries of age-related biomarkers for frailty (24)(39). With the aging of the population and the increase in the number of very old persons, the progression to frailty from healthy aging, particularly in the oldest-old (90 years and above), appears to be an important and timely reality. This is confirmed in our clinical practice, particularly in the frailty clinic of the Gérontopole of Toulouse (40). A possible concept for age-related frailty is presented in the study by Angioni et al. (41).

## Frailty and chronic disease

While aging itself is commonly accepted as the main risk factor for chronic diseases (42), new findings suggest the reciprocal may also be true (43). There are well-known links between specific age-related chronic diseases (e.g. diabetes, cancer, CVD) with many hypotheses explaining their associations (44, 45).

The relationship between frailty and chronic disease is complex and remains unresolved (9, 26). Whether multimorbidity is a precursor of frailty has rarely been studied and the evidence is therefore scarce (26). Although it is agreed that frailty may lead to multimorbidity, it is also widely believed that multimorbidity is a predictor of frailty (46, 47) (48). Multimorbidity is defined by the presence of two or more chronic disease (49, 50) and tends to arise earlier in life before the onset of frailty (51). Moreover, the associations between frailty and multimorbidity might probably depend on the operational definition of frailty. There are plenty of tools being used for measuring frailty (4, 5, 52) and multimorbidity (53, 54, 55), and these tools are probably not selecting the same profile of individuals. In the context of frailty, the guidelines for the management of multimorbidity are rare (56), with most evidence focusing on single disease management. This poses a great challenge for geriatricians and treating physicians, and increases suspicion towards the role of multimorbidity in the progression of frailty.

In 2018, Hanlon et al. found that frailty was significantly associated with multimorbidity (prevalence 18% [4,435/25,338]) in those with four or more long-term conditions (49). In their findings, multiple sclerosis, chronic fatigue syndrome, connective tissue disease, diabetes and chronic obstructive pulmonary disease were the main diseases associated with frailty. Moreover, comorbidities such as congestive heart failure, myocardial infarction, rheumatoid arthritis, peripheral vascular diseases, diabetes and hypertension have been seen to increase the risk for frailty (37). Vetrano's meta-analysis (26) similarly found that the prevalence of frailty among multimorbid individuals was 16%. Multimorbidity was associated with frailty in pooled analyses (odds ratio = 2.27). Three longitudinal studies suggested a bidirectional association between multimorbidity and frailty (46, 57, 58).

In conclusion, a single or multiple chronic diseases may trigger the onset of frailty. The geroscience hypothesis is that aging is the major modifiable risk factor for chronic diseases (43). Further studies are needed in order to clarify the relationship between frailty and comorbidity in order to work on aging.

## Frailty and Geroscience

Biological — but not chronological — aging is the major risk factor for age-related diseases, as well as frailty and loss of resilience and function. Recent studies have confirmed that biological aging is malleable and it can be delayed in mammals (42). However, in humans the role of aging on the onset of clinical events has only been studied using chronological age. The field of geroscience aims to determine the mechanisms by which biological aging accelerates morbidity, as a way to identify novel therapeutic targets to maintain function and postpone age-related diseases. The aim is to use the pillars/hallmarks of aging (42, 59) to identify molecular targets that delay the aging process, as a way to delay multiple diseases at once. Examples include the use of senolytics (42, 60), inhibitors of nutritional sensing (61), NAD+ precursors (62) and other interventions based on the pillars/hallmarks of aging. Among the different biomarkers of frailty, only inflamaging appears to be a convincing candidate as a biomarker of frailty (63). Application of geroscience principles in the clinic would be strongly abetted by availability of information on the frailty status of the individual, as well as the origin of that frailty, either age-related or disease-driven. It is surmised that geroscience approaches will be most effective in those individuals whose frailty (or pre-frailty) is driven by aging, rather than disease.

While it is currently possible to measure frailty and disease in the clinic, measurements of biological aging are only in their infancy. Several physiology-based approaches are being proposed and tested (64), but molecular measurements of the rate of aging are less well-developed, with the most promising being the epigenetic clocks (64). It is becoming therefore increasingly urgent to identify with further granularity the phenotype of aging and its relation to frailty (65). There is precedent for advancing both science and health care by more granular definition of phenotypes. For example, for a longtime in clinical practice, age-related cognitive decline was considered as part of a natural evolution; however, refinements of how we define Alzheimer's disease have led to the identification of prodromal syndromes such as mild cognitive impairment (MCI). We expect that a similar pathway will probably happen for age-related frailty.

## Conclusion

With the increase of very old subjects in our clinical practice, we often observe that these very old subjects (>90 years of age) do not present any comorbidity, which probably explains why they live longer than the average. However, at some time, they become frail and we need to understand why. The Inspire program (66, 67, 68, 69), is planning to work on the links between biological age and “age-related frailty”. Future recommendations for frailty management and prevention will probably have to be adapted to these two etiological pathways, age-related frailty and frailty related to diseases (70), leading to the same clinical condition. We think that aging is in conclusion the main risk factor for frailty (through chronic diseases or not) and consequently aging should be the main target for interventions.
